# Facial expression as a potential measure of both intent and emotion

**DOI:** 10.1038/s41598-018-35905-3

**Published:** 2018-12-04

**Authors:** Irene Camerlink, Estelle Coulange, Marianne Farish, Emma M. Baxter, Simon P. Turner

**Affiliations:** 1 Animal Behaviour & Welfare, Animal and Veterinary Sciences Research Group, Scotland’s Rural College (SRUC), West Mains Road, Edinburgh, EH9 3JG UK; 2 Institute of Animal Husbandry and Animal Welfare, Department of Farm Animals and Veterinary Public Health, University for Veterinary Medicine Vienna, Veterinärplatz 1, 1210 Vienna, Austria

## Abstract

Facial expressions convey information on emotion, physical sensations, and intent. The much debated theories that facial expressions can be emotions or signals of intent have largely remained separated in animal studies. Here we integrate these approaches with the aim to 1) investigate whether pigs may use facial expressions as a signal of intent and; 2) quantify differences in facial metrics between different contexts of potentially negative emotional state. Facial metrics of 38 pigs were recorded prior to aggression, during aggression and during retreat from being attacked in a dyadic contest. Ear angle, snout ratio (length/height) and eye ratio from 572 images were measured. Prior to the occurrence of aggression, eventual initiators of the first bite had a smaller snout ratio and eventual winners showed a non-significant tendency to have their ears forward more than eventual losers. During aggression, pigs’ ears were more forward orientated and their snout ratio was smaller. During retreat, pigs’ ears were backwards and their eyes open less. The results suggest that facial expressions can communicate aggressive intent related to fight success, and that facial metrics can convey information about emotional responses to contexts involving aggression and fear.

## Introduction

Facial expressions convey information on physical sensations, for example pain, as well as emotional states and intent. It has been debated whether facial expressions evolved to communicate intention (motive-communication approach^1^), for example to attack, or whether it is the display of emotion as a consequence of inner state (emotion-expression approach^2^)^3–5^. In humans, the occurrence of these two models was evaluated by human ratings on the display of feelings, intentions or action requests in images showing facial expressions^5^. The facial expressions of fear, sadness, happiness, disgust and surprise were interpreted as the display of an emotion whereas anger was rated as a behavioural intention^5^. To date, the two lines of thought remain largely separated^6^, including in animal studies, although it has been theoretically suggested that they could exist alongside each other^7^. In behavioural ecology the focus is predominantly on facial expressions as signals of intent^1,6,7^ whereas in animal sciences facial expressions are being studied as a reflection of pain (e.g. rodents^8^; sheep^9^; pigs^10^) or as a measure of emotional state (reviewed by^11^). In the current study we integrate these two fields by assessing facial expressions during animal contests using a behavioural ecology approach with applied relevance to animal welfare.

During contests individuals can signal their intent to attack or withdraw^12^, but they may also experience strong emotions of anger or fear^11,13^. Agonistic encounters between animals can be costly and may even result in death (reviewed by^14^). Minimizing the costs of aggression is therefore important. Signalling of intent can minimize such costs, for example by signalling submission to avoid further costs^15,16^. Signals of intent will occur prior to the action or behaviour it represents^15^. If signals are not counteracted by the receiver, then the signaller may eventually perform the intended action or behaviour^14,15^. An aggressive signal may therefore predict an attack or the next level of escalation^14,17^. For example, if threat behaviour is not followed by a submissive response from the receiver then the signaller may commence stronger signals and eventually attack. In turn, attack initiation increases the likelihood of winning a contest when the attack is not retaliated^18^. Thus, if a facial expression is an honest signal of intent, it should reliably predict the respective future behaviour or outcome. Our first aim was to investigate how facial expression prior to the occurrence of aggression relates to the onset of aggression and its outcome.

Aggression between individuals is a considerable welfare issue in domestic animals^19^. Besides the costs of aggression in terms of injuries, the emotional state of the animal will be negatively affected^19^. Emotions are to date an integral part of animal welfare^20,21^. Hereby facial expressions are increasingly explored in the context of animal welfare as a non-invasive method to obtain quantitative measures that convey information about emotion^11^. An emotion will, at least in non-human animals, be expressed at the moment or closely after the experience of a change in the psychological state^22,23^ (in contrast, humans may make deliberate facial expressions for social or cultural reasons and thus may not directly reveal their emotional state^24^). Facial expressions conveying information about the emotional state will therefore relate to the animal’s perception or emotional valence (i.e. affect) of the situation at that moment. Our second aim was to quantify differences in facial metrics during contexts related to aggression and fear. If facial expressions reflect emotions, then different facial metrics would be shown during or after the behaviours related to the specific emotions, e.g. attack behaviour being related to anger/aggression and flight behaviour being related to fear.

The above aims were studied in dyadic contests between pigs. Animal contests were carried out within a project that utilized game theory models as advocated in behavioural ecology, as well as applied animal welfare aspects of aggression between pigs^25^. Pigs are facially communicative^10^ and have little hair coverage compared to most other species, making them an ideal species in which to study the emotional significance of facial expressions. This study captured pigs’ facial expression prior to the occurrence of aggression, during aggression, and during retreat from being attacked (a situation likely to elicit fear) and related the facial metrics to the behaviour. Regarding the first aim, we hypothesized that pigs signal their intent to attack through a facial expression prior to aggression. Regarding the second aim we hypothesized, based on studies of facial expression of emotion in other species^9^, that during aggression the snout will show a more pronounced nose wrinkle (pigs^10^) and the upper lip will be raised (mice^26^; humans^27^), whereas during retreat we expect ears to be held backwards (as shown in horses^28,29^, sheep^30^, dogs^31^, mice^26^ and pigs^32^). We studied this by analysing 572 images for facial metrics.

## Results

**Figure 1:**
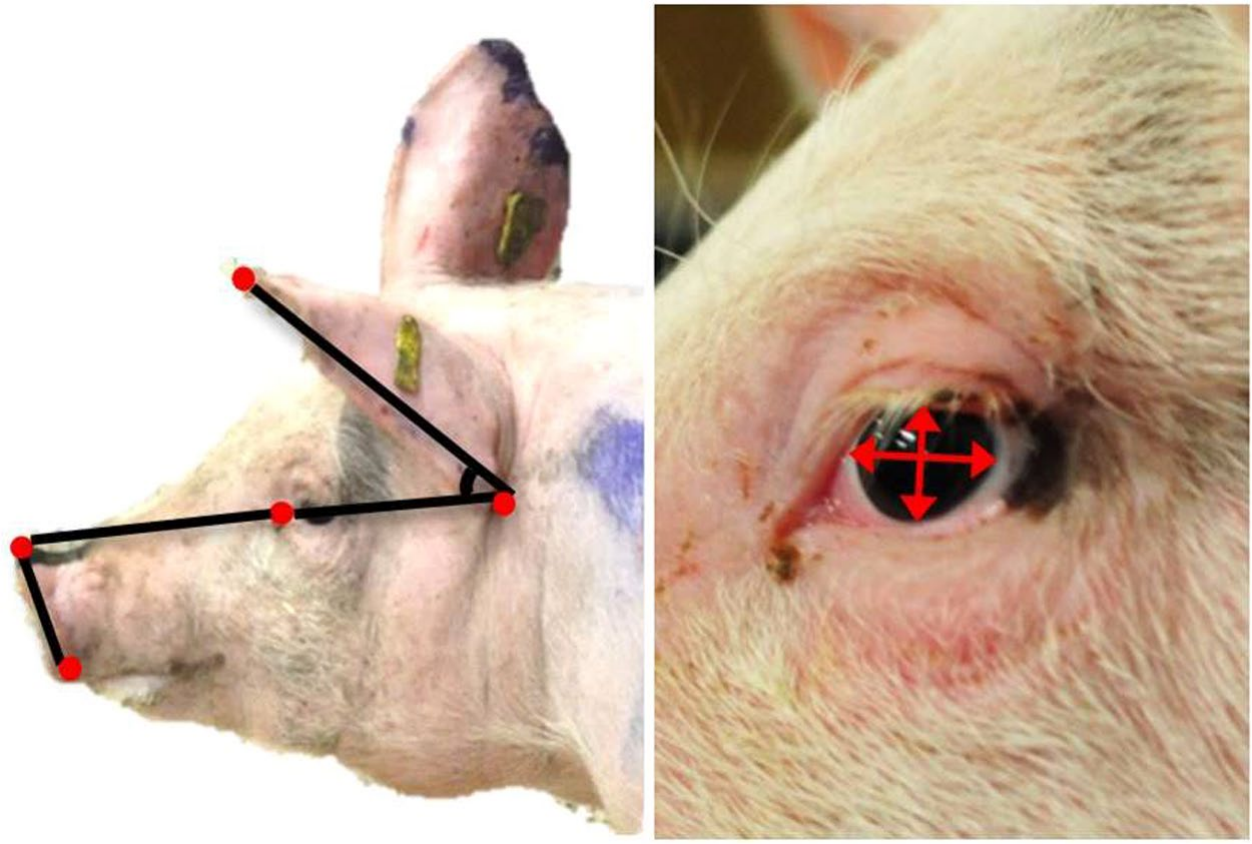
Facial measurements. Red dots indicate the fixed points from where measures were taken whereas black lines indicate the measurement of eye-nose length, nose disk length, and ear angle (**A**). (**B**) shows the measurement of the eye ratio (height/width).

Images of the head were taken in the phase prior to any agonistic behaviour (pre-agonistic), during aggression and during retreat from being attacked (full description in Table 1) within the course of a dyadic contest between unfamiliar pigs (n = 38 pigs). The pre-agonistic situation was short and resulted in 80 usable images (~3 frames/pig). During aggression 264 images were obtained (~7 frames/pig) and during retreat from aggression 228 (~7 frames/pig). From each image the ear angle, snout ratio (length from eye to nose/length from top to base of nose disk; Fig. 1a) and eye ratio were calculated (height of eye/width of eye; Fig. 1b). The profile of the face in the image (full- or quarter profile) affected the facial metrics and was retained in all models to account for this (influence of profile on ear angle: *F*_1,475_ = 22.29; *P* < 0.001; on eye ratio: *F*_1,485_ = 6.51; *P* = 0.01; and on snout ratio: *F*_1,489_ = 20.94; *P* < 0.001).

**Table 1:** Description of circumstances in which images where obtained from videos.

Situation	Description
Pre-agonistic	Focal animal has entered the contest arena and has not yet made contact with the opponent. No aggression has yet occurred.
Aggression	Focal animal initiates an aggressive act (push or bite) or retaliates to an aggressive act. In the case of biting, the frame just before or after the bite was selected so that the mouth was not fully opened.
Retreat	Focal animal withdraws from aggressive act, either by showing a head-tilt, turning the body 180° away from the attacker, or by fleeing from the attacker.

### Preparing for attack

**Figure 2:**
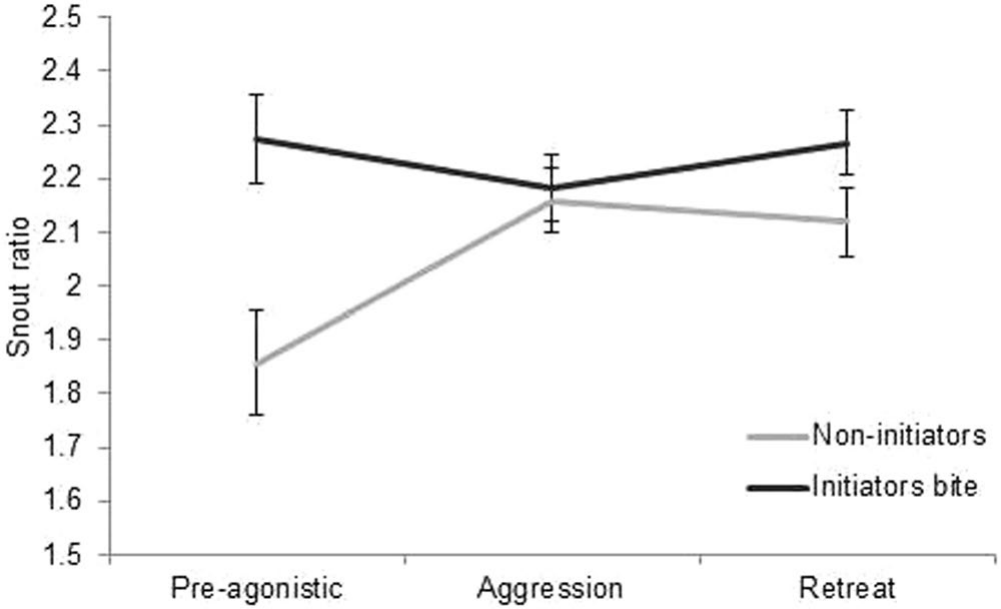
Snout ratio for opponents that initiated the first bite (i.e. first attack) and for opponents that did not initiate the first attack, during the phase prior to agonistic behaviour, during aggression and during retreat. n = 38 pigs (535 images).

Signals of intent were investigated by relating the facial metrics in the phase prior to agonistic behaviour (pre-agonistic) to the initiation of nose-contact and aggression. The initiator of the first nose-to-nose contact had, prior to this contact, a larger eye ratio, meaning that its eyes were more open (eye ratio initiator contact 1.05 ± 0.03; non-initiator 0.99 ± 0.03; *F*_1,497_ = 4.31; *P* = 0.04). The ear angle or snout ratio did not differ regarding subsequent nose-to-nose contact (*P* > 0.10). The initiator of the first bite had a greater snout ratio than the opponent that did not initiate aggression after the pre-agonistic situation (Fig. 2; *F*_1,535_ = 12.92; *P* < 0.001). A greater snout ratio signifies either an increase in the eye-nose length or a decrease in the height of the nose disk, making the snout more elongated. During aggression the bite initiator and non-initiator did not differ in their snout ratio (Fig. 2), revealing a tendency for an interaction between bite-initiation and context on snout ratio (*F*_2,535_ = 2.37; *P* = 0.09). The ear angle and eye ratio did not differ for bite initiators. The facial metrics prior to aggression were unrelated to the initiation of the first fight (all *P* > 0.10).

### The face of victory

To test how potential signals of intent would affect contest outcome, the relationship between the facial expression prior to aggression and during aggression on contest outcome was examined. Prior to the occurrence of agonistic behaviour the eventual winners showed a non-significant tendency to have their ears orientated more to the front than eventual losers (ear angle winners during pre-agonistic situation 81.3 ± 4.9; losers 89.9 ± 4.7; *F*_1, 482_ = 1.72; *P* = 0.09), but they did not differ in snout ratio or eye ratio (*P* > 0.10). During aggression and retreat winners and losers did not differ in ear angle, snout ratio or eye ratio (all *P* > 0.10). Losers may receive more bites than winners and the resulting skin lesion may be painful, which in turn may affect facial expression. Pigs with more skin lesions on their body had their ears oriented more to the front (*b* = −0.03 ± 0.01/skin lesion; *F*_1,482_ = 6.47; *P* = 0.01). Skin lesions did not affect the eye ratio or snout ratio (all *P* > 0.10). Moreover, there was an effect of previous experience of losing. Pigs which had lost a dyadic contest three weeks earlier had, throughout the contest, a larger eye ratio than opponents that had a previous experience of winning a contest (prior loser 1.05 ± 0.03; prior winner 0.99 ± 0.03; *F*_1,497_ = 4.55; *P* = 0.03). Prior winner-loser experience did not influence the ear angle or snout ratio (*P* > 0.10).

### Facing the situation

**Figure 3:**
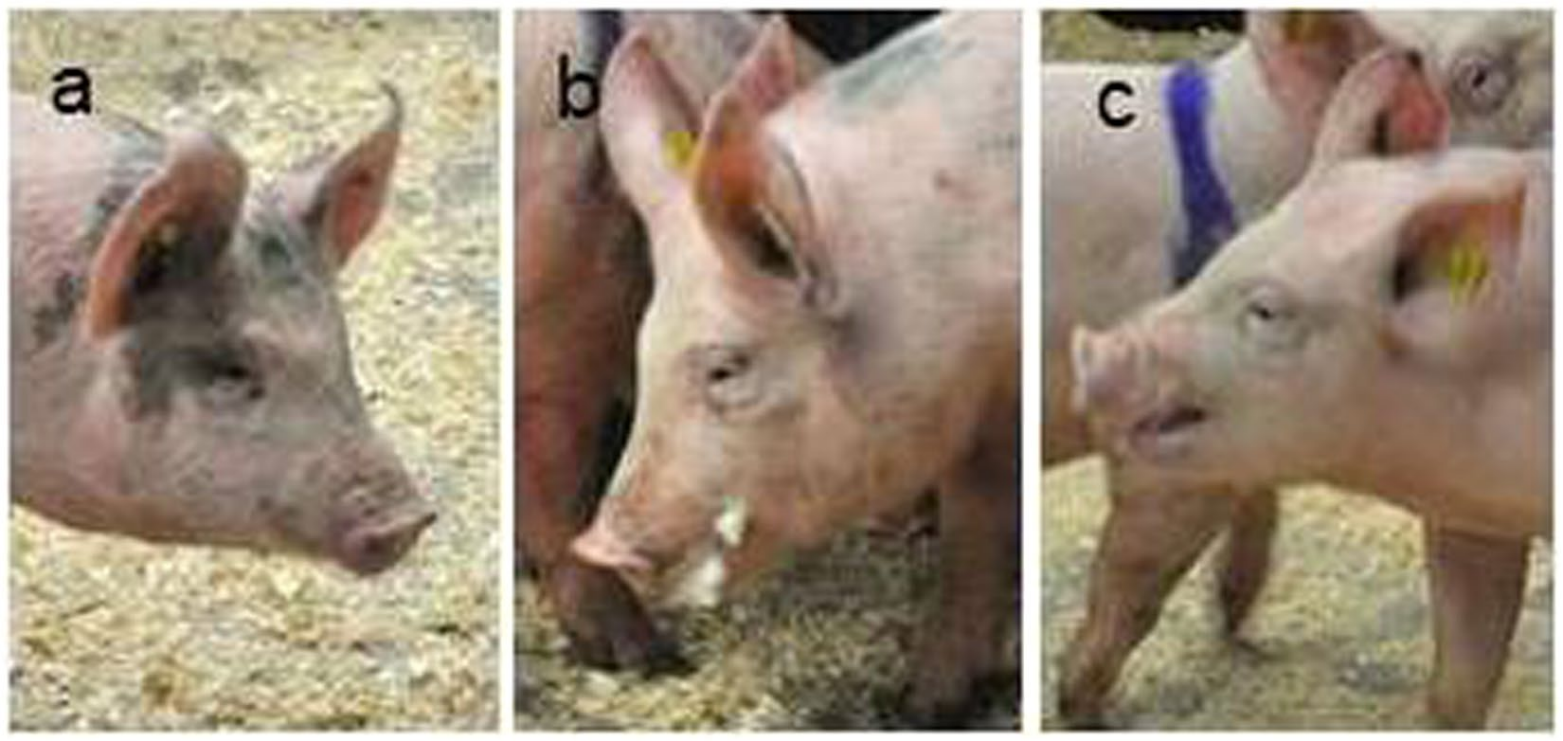
Facial expressions in three different scenarios. (**a**) pre-agonistic situation; (**b**) during aggression; and (**c**) during retreat from attack. The images are representative of the average and are chosen to reflect the statistical means.

**Figure 4:**
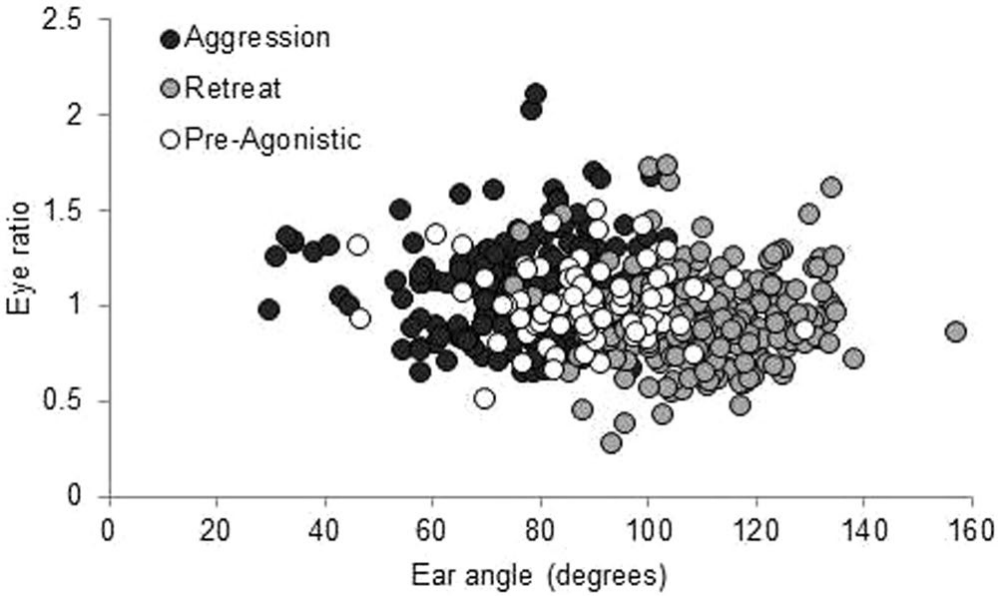
Ear angle in relationship to the eye ratio. Ear angle during the pre-agonistic situation, during aggression and during retreat from attack.

Facial expression prior to agonistic behaviour, during aggression and during retreat differed significantly from each other for the ear angle and eye ratio (Table 2). Figure 3 gives a representation of the average facial expressions during the three contexts and the images are selected to reflect the statistical means. The facial expression during retreat (i.e. withdrawal from attack) was most distinct, with the ears being more backward orientated (Fig. 4) and the eyes slightly closed (Table 2). More examples of images during these situations are provided in the Supplementary files (Supplementary Figs S1; S2; S3).

**Table 2:** Facial metrics prior to aggression, during aggression and during retreat from aggression. Values are LSmeans with SEM.

Facial metrics	Pre-agonistic	Aggression	Retreat	Test statistics
Eye ratio	1.07 ± 0.04^a^	1.02 ± 0.03^a^	0.98 ± 0.03^b^	*F*_2,497_ = 4.91; *P* = 0.008
Snout ratio	2.08 ± 0.06^a^	2.16 ± 0.05^a^	2.17 ± 0.05^a^	*F*_2,535_ = 1.39; *P* = 0.25
Ear angle (in degrees °)	85.6 ± 4.13^a^	88.3 ± 3.72^ab^	93.6 ± 3.72^c^	*F*_2,472_ = 5.94; *P* = 0.003

^a,b,c^Letters lacking a common superscript differ by *P* < 0.05.

The recorded facial metrics were not influenced by the opponents’ sex, body weight or body conformation as measured by ponderal index. Images taken from the left side of the face did not differ from images taken from the right side of the face, indicating, in this particular case, no lateralization of facial metrics.

## Discussion

Facial expressions have been discussed as being an expression of emotional state or signal of intent, but these theories have largely remained separated in animal studies. This study integrated these lines of thought (as suggested in^7^) using an interdisciplinary approach combining theories from behavioural ecology with animal welfare science. Facial metrics prior to agonistic behaviour related to the initiation of an attack, indicating facial expression as an honest signal of intent^16,17^. Facial metrics during retreat from aggression differed from those prior to and during aggression, supporting the view that facial expressions can convey information about emotional state. Although all three contexts could be experienced as negative, the facial metrics within these negative contexts show differences between them, likely reflecting states of anticipation or ‘action readiness’^26^, aggression and fear.

**Figure 5:**
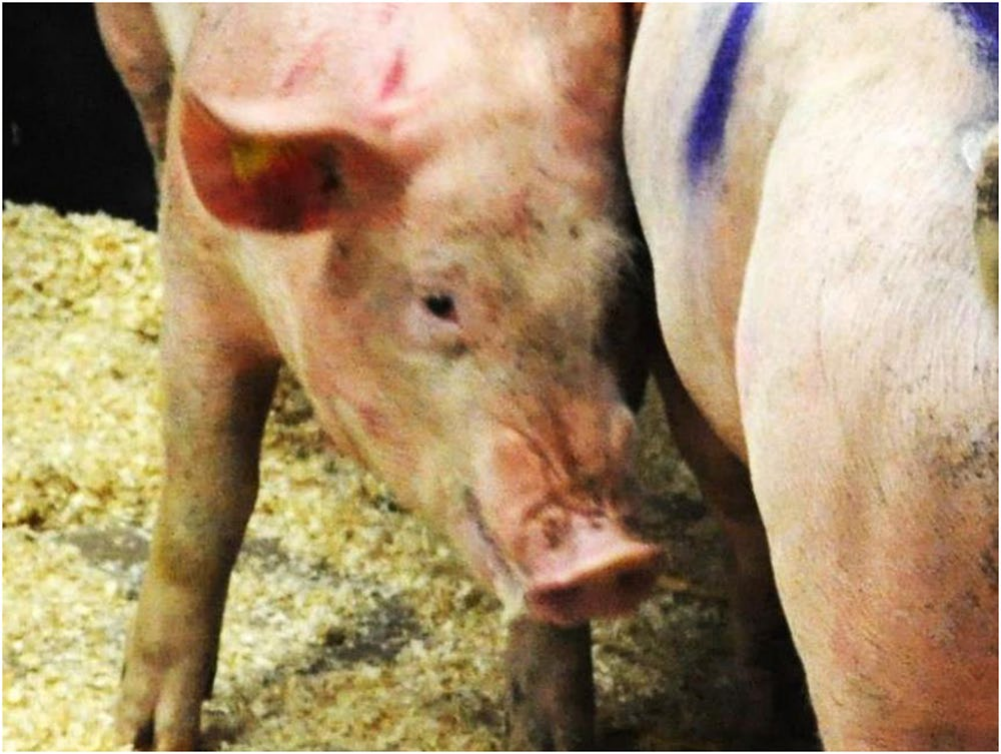
Nose snarl during fight. The nose snarl is characterized by pulling up the upper lip (*levator labii superioris*) (or *levator labii superioris alaeque nasi* muscle in humans); in pigs: nasolabial levator, attached to the upper lip) and tightening of the caninus muscles (*levator anguli oris*).

Our first hypothesis that pigs would signal their intent to attack through a facial expression prior to aggression was confirmed by the current study. Prior to the onset of aggression, eventual initiators of the first attack (bite) showed a greater snout ratio than non-initiators, corresponding to a more elongated snout. Pigs may snarl to signal aggression (Fig. 5). During this ‘snarling’ a strong nose wrinkle appears (which is a Facial Action Unit related to aggression in some species; humans^33,34^, canids^35^) and the upper lip is raised^33^. This may coincide with an additional raise at the location of the incisor which reveals the teeth^35,36^. In *Sus scrofa*, the wild ancestor to the domestic pig, this would reveal the tusks if present (for examples see Supplementary Fig. S2). Tusks are, by some members of the taxa, used as weapons in fights^36^. Direct observation of the facial metrics during a snarl would be needed to confirm whether this is the case for the current data.

Subsequent winners showed a non-significant tendency to have their ears more forward-oriented than losers in the phase prior to the occurrence of aggression. This may be indicative of motivation or intent to win or might be a trait that itself directly contributes to success during the subsequent fight. In humans, facial expressions can convey information not only about motivation, but also ability, as the expression when angry can contain information about the physical strength of the person^27^, and thus their fighting ability, also termed resource holding potential^37^. The tendency for the forward orientation of the ears may be associated with a state of heightened attention, motivation, confidence or aggression^11,38^, which suggests that winners had a greater readiness to engage in aggression than losers^26^. Susskind and colleagues^39^ proposed that facial expressions are adaptive action tendencies serving to prepare the organism for a situation so that appropriate action can be taken. It may, however, be that the phase prior to aggression already elicited a heightened state of alertness or fear. Animals were well habituated to the test situation but had experienced a previous contest in the arena three weeks earlier. A previously negative experience of losing (which may result in so called winner-loser effects^40^) did indeed relate to a greater eye ratio (i.e. eye widening) throughout the contest, which can indicate fear^11,41^.

Our second aim was to quantify differences in facial metrics during contexts known to elicit emotions of aggression (attack behaviour) and fear (flight behaviour). Our hypothesis that during aggression the pig would show a smaller snout ratio whereas during retreat the ears would be held backwards was only partly demonstrated. The three contexts resulted in differences in the eye ratio and the orientation of the ears but not in snout ratio.

During aggression pigs had their ears erect and their eyes more widely opened than during retreat, but the snout ratio did not differ from those in the other contexts studied. In other species it has been found that during aggression the upper lip may rise (humans^42^). As described above, differences in the snout ratio were related to bite initiation. Bite initiators had a greater snout ratio, suggesting that the facial metrics may already be affected early on in agonistic interactions. In addition, laboured breathing during a fight and preparation for biting, both of which require opening of the jaws, might affect the snout ratio beyond any potential effect of emotional state. Images in which the animal was biting were excluded but such a scenario could, to some extent, influence the facial metrics.

During retreat from being attacked, pigs had their ears orientated backwards and their eyes slightly closed. The backward position of the ears is in line with facial expressions reported in other species during fear or pain, including horses^28,29^, sheep^30^, dogs^31^, mice^26^ and pigs^32^. In pigs the backward orientation of the ears has previously been related to negative experiences, even in the absence of pain^32^. The backward position of the ears in the present study is therefore unlikely to result purely from the need to hear a pursuing attacker, but indicates that the occurrence of being attacked (i.e. bitten by the opponent) is aversive.

Pigs closed their eyes slightly during retreat. In other species partial closing of the eyes, often referred to as orbital tightening, has been related to pain^8,43,44^ and is one of the main facial action units comprising the grimace scales developed for assessing pain in different species, including pigs^10^. For expressions relating to fear the upper eye lid is commonly raised whereas the lower eye lid is tightened^45^, which can result in an increased visibility of the eye white, as for example recorded in cows during fear-inducing situations^41^. However, the use of the ‘upper lid-raiser’ and the ‘lid-tightener’ Facial Action Units (FACs), which provide information on the eye, has been questioned as they are both of relevance during aggression^27^. Sell and co-authors^27^ hypothesized that during aggression, the lid-raiser may operate in response to surprise whereas partially closing the eye with the lid-tightener may improve focus. In mice, eye tightening has been observed during a social encounter without aggression; when exposed to a rat; and during attack when mice defended their territory against an intruder^26^. Partially closing the eyes can also be an instinctive response to protect the body against harm^26^; for example non-human primates and mice close their eyes and flatten their ears when startled to reduce exposure of sensory organs^26,39^. This may be particularly relevant for pigs as attacks (bites) are mainly targeted towards the face. We are therefore cautious in interpreting the eye ratio.

Facial expression has mainly been studied by using the Facial Action Coding System (FACS)^46^ and grimace scales for pain^8^. FACS has not yet been adapted for pigs and a grimace scale for pain in piglets has only recently been described^10^. As such, we chose to record the main facial metrics that have previously shown to relate to the expression of aggression and fear^11^. Limiting the number of facial metrics allowed the evaluation of a greater number of images and provided guidance for further research on facial expressions. The current data suggest that the information content of facial expressions is similarly rich in pigs as in other species. It is therefore likely that the communication function of facial expressions is significant and, to date, under-appreciated both in behavioural ecology and in animal welfare science.

Darwin already in 1872 observed that emotions are accompanied by facial expressions^34^. That does not, however, imply that a facial expression always indicates an emotion per se. Although facial expressions have predominantly been studied in the context of emotions, this does not make a certain facial expression a stand-alone indicator for an emotion. As shown here, facial expressions can also be a signal of intent depending on when the facial expression occurs. They can also serve to protect sensory organs. If the facial expression follows an emotion^23^ then this should be interpreted alongside behavioural data, such as applied here for aggressive behaviour and flight behaviour, and/or physiological data to underpin any conclusions about the animal’s emotional state. With the growing interest in the use of facial expressions to interpret animal welfare states we recommend further research on their meaning and the use of correlated measures to support claims. Quantitative data on facial metrics during both positively and negatively valenced situations, and the measurement of physiological and cognitive parameters to cross-validate facial expressions with other qualitative and quantitative measures of emotional state, could thereby be a great asset to the fields of behavioural ecology and animal welfare science^11^.

## Conclusion

Facial expression has long been treated either as signal of intent or emotional state. In dyadic contests between pigs, facial metrics prior to the onset of agonistic behaviour related to the subsequent initiation of an attack, indicating that facial metrics can be a signal of aggressive intent. Facial metrics during and after retreat from being attacked differed significantly from the facial metrics prior to and during aggression, with the facial metrics during retreat being similar to what in other species has been related to fear. In conclusion, this study shows that facial expressions can be a signal of intent as well as a reflection of emotional state.

## Methods

### Ethics

This study was part of a larger project on aggression between pigs^25^. The trial was carried out in accordance with the recommendation in the European Guidelines for accommodation and care of animals, UK Government DEFRA animal welfare codes, and adhered to the ASAB (Association for the Study of Animal Behaviour) guidelines. All procedures were approved by SRUC’s Animal Ethics Committee (no. ED AE 21-2014) and the UK Government Home Office under the Animals Scientific Procedures Act 1986 (project licence PPL60/4330). Strict end-points were in place for the contest but none of the thresholds (escape attempt; continuous vocalization; mounting behaviour; or a time limit of 20 min) were reached in the current study. Animals had no injuries other than skin lesions as a result of bites, and such lesions healed within 24 h without the need for medical intervention.

### Animals and treatments

Commercial crossbred pigs (Large White × Landrace dam × American Hampshire sire) were raised at the SRUC research farm (U.K.) in three subsequent batches. A subsample of 38 pigs (26 males; 12 females) was included in the study on facial expression. Pigs were weaned at four weeks of age after which they remained in their litter group. Pens measured 1.9 × 5.8 m (~1.1 m^2^/pig) and had a solid floor and straw bedding. Pens were cleaned daily and replenished with fresh straw and dry pelleted food and water were available ad libitum. At seven weeks of age, animals were weighed and their circumference and crown-rump length was measured to calculate a ponderal index (index of weight in relation to length, calculated as [body weight]/[crown-rump measurement]^3^). Ponderal index is a measure similar to the Body Mass Index and was included here as facial metrics might change with a higher index.

### Contests

At 13 weeks of age pigs were staged into pairwise contests for another research question on aggressiveness^25^. Opponents were unfamiliar to each other and were matched randomly with regard to body weight and sex. When unfamiliar pigs meet this commonly results in aggressive interactions within several minutes. Pigs had previously been habituated to walking to the contest arena individually to avoid a fear response obscuring the interaction between opponents. Also, pigs had had a similar contest situation three weeks earlier (with a different opponent), the outcome of which was included in the statistical model. Just before entering the test arena, pigs were weighed and, while in the crate, a few drops of blood were obtained from the ear vein by pricking it using a capillary blood lancet with a flat blade. This was for a purpose separate to the current study. Immediately following this pin-prick procedure the ear was cleaned to ensure no traces of blood were present. During the contest opponents entered the arena simultaneously from opposite directions. The contest arena was 3.8 × 2.9 m and had a solid floor covered with a thin layer of wood shavings. Pigs had a spray marked number on their back for recognition. The term ‘contest’ here refers to the moment from the opponents entering the arena until a clear winner was present (described below). Contests included a complex sequence of behaviours, involving alternating phases of escalation and de-escalation whereby retreat can also occur as an interlude before recommencing reciprocal fighting (for details see^47^). During the contest it was noted which individual initiated the first nose-to-nose contact (non-agonistic behaviour), the first unilateral bite (bite without retaliation of the opponent) and the first fight (mutual engagement in aggressive behaviour including bites retaliated by the opponent). Contests were ended when a clear winner was apparent. This was when one of the pigs retreated, shown by the clearly distinguishable head-tilt movement^47^ when the subordinate rapidly turned away from the other, and did not show any aggression for 2 min after its last head-tilt retreat. Aggressive behaviour from the winner towards the loser after retreat was recorded as bullying behaviour of the winner. Contests were also ended when an end-point was reached (a maximum duration of 20 min; sustained fear response; or mounting behaviour). The number of skin lesions as a result of bites was counted just before and just after the contest by a single observer. For details of the test see^25^.

A camera with a wide angle lens (Canon Legria HFM52, Canon Inc., Tokyo, Japan) was fixed on a tripod placed just outside one corner of the contest arena and was adjusted as close as possible to the eye-height of the pigs. Contests were filmed for the full duration. Only contests which lasted at least 2 minutes and included aggressive behaviour were used for the analyses on facial expression.

### Extraction of images

Videos were played frame by frame with the free software Avidemux (Open Source, version 2.6.14, Windows 32 bits), which allows a precise selection of pictures and their extraction as image files. Images were obtained from three different scenarios all taken during the period that the opponents were in the arena together: during a pre-agonistic situation, during aggression, and during retreat from aggression. The description of these contexts is given in Table 1. Images for the aggressive scenario were taken from several phases of escalation. Where possible, frames during aggression were selected just before or just after biting, when the mouth was not fully opened. Images for retreat were taken when shown during an on-going fight as well as at the end of a fight (final withdrawal). Only full profiles and quarter (or three quarter) profiles were selected for analysis. For every image it was specified whether it was a full or quarter profile (full profile n = 262 images; quarter n = 310) and whether the image was from the left or right side of the face (left n = 271; right n = 301). Frames where discarded when the image quality was low or when the image was deformed due to rapid movement. The selection of two consecutive frames was avoided where possible, but 11 consecutive frames had to be selected to obtain the minimum number of good quality images per animal (three during the pre-agonistic situation; five during aggression; and three during fear).

The majority of the pigs had naturally upright ears (33 out of 38 pigs). Measurements of the ear angle were excluded for five pigs that did not have erect ears. Pigs were white or white with black patches and two contests had to be discarded because the position of the dark patches around the eyes limited precise measurements. Images were extracted and data were collected by one person.

### Facial measures

We decided to take quantitative measures of the facial features rather than using a qualitative grimace scale or qualitative behavioural assessment (QBA^48^) as the presence of bite marks (skin lesions) and reddened skin due to intense activity and fighting may bias observers in their evaluation of the facial expression. Facial measures were made with the free software ImageJ version 1.48 (Wayne Rasband, National Institutes of Health, USA). This software enables automatic calculation of the distance between two points and the angle between two lines. In order to ensure repeatability, measures were made from fixed points on the face. These were the top and base of the nose disk, the top and base of the ear and the inner corner of the eye (lacrimal caruncle) (Fig. 1). From these points the length from the eye to the top of the nose disk, the distance from the top to base of the nose disk, and the ear angle were obtained. Because images were taken from different distances to the camera the actual distance between facial features was not used but instead a snout ratio and eye ratio were calculated from the fixed points. The snout ratio was calculated as: [length from eye to nose]/[length from top to base of nose disk] (Fig. 1a). The eye ratio was calculated as: [height of eye]/[width of eye] (Fig. 1b). Measures on the eyebrow could not be obtained due to the colouring of the skin in combination with the quality of the images.

### Statistical Analysis

Data were analysed with the SAS version 9.3 (SAS Institute Inc, Cary, USA). The dependent variables were the snout ratio, eye ratio and ear angle. These measures showed a normal distribution of the residuals and were accordingly analysed in generalized linear models (MIXED Procedure). A repeated statement (pig ID) was included to account for repeated observations per pig. The litter group was included as a random effect to account for genetic similarities between pigs and dyad number was included to account for dependence of opponents within the same contest. The fixed effects were the situation in which the image was taken (pre-agonistic/aggression/retreat), sex (male/female), profile angle (full profile or quarter or three quarter profile), lateralization (profile was viewed from the left or right), body weight at 13 wk of age, ponderal index at 10 wk of age, contest outcome of the current contest (winner/loser contest 2), contest outcome of the previous contest (winner/loser contest 1) and the number of skin lesions. The initiation (yes/no) of the first nose-to-nose contact, the first bite and the first fight were also included. Relevant interactions between a variable and the situation (pre-agonistic/aggression/retreat) were retained in the model if significant. Variables with a *P*-value > 0.10 were stepwise removed from the model while assessing the Akaike information criterion (AIC) for the best model fit. The profile angle significantly affected the facial metrics and was retained in all the models as a correction factor. Results are presented as LSmeans with standard error. *P* values below 0.05 were considered significant whereas values >0.05 but <0.10 were reported as tendencies with their actual value. Any value above was reported as >0.10.

## Electronic supplementary material

Supplementary file S1, S2 and S3

## Data Availability

The datasets generated and analysed during the current study are available from the corresponding author on reasonable request.
