# Supplementary material for: Facial expression as a potential measure of both intent and emotion
Source: Sci Rep. 2018 Dec 4;8:17602. doi: 10.1038/s41598-018-35905-3 (PMC6279763; doi:10.1038/s41598-018-35905-3)

Facial expression as a potential measure of both intent and emotion

Irene Camerlink, Estelle Coulange, Marianne Farish, Emma M. Baxter, Simon P. Turner

Supplementary file

Figure S1. Examples of facial expressions during pre-agonistic phase.

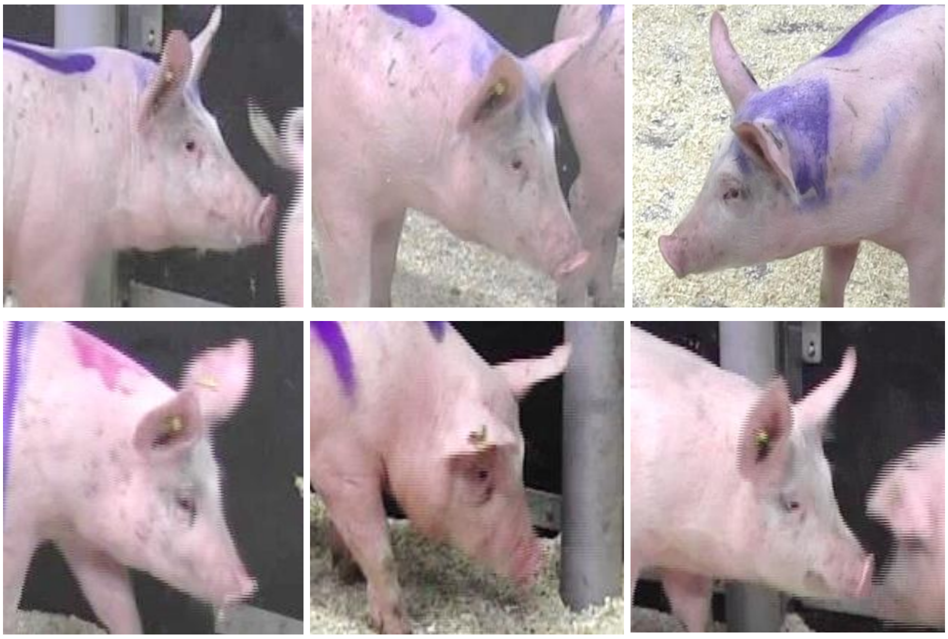

Figure S2. Examples of facial expressions during aggression.

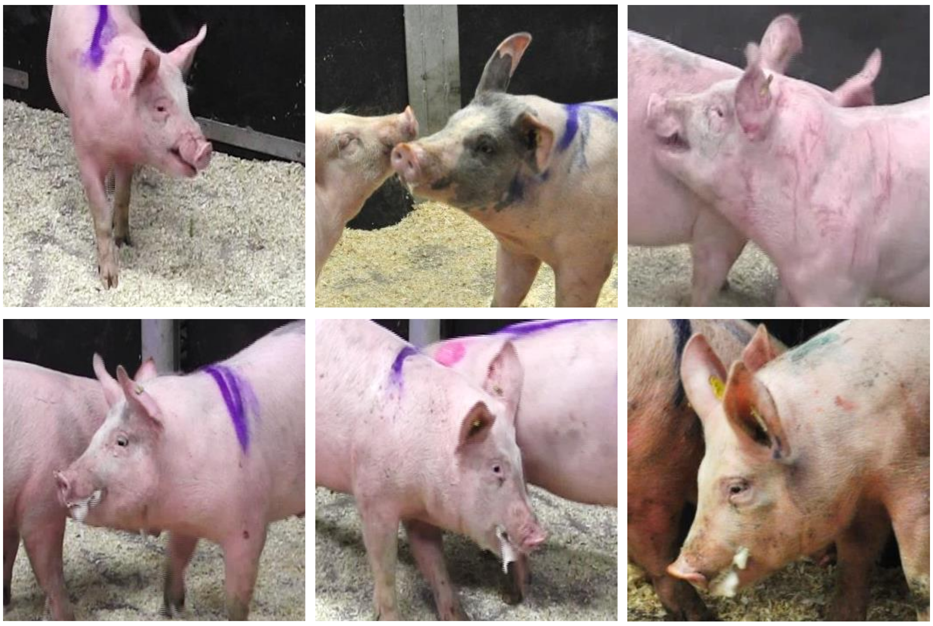

Figure S3. Examples of facial expressions during retreat.

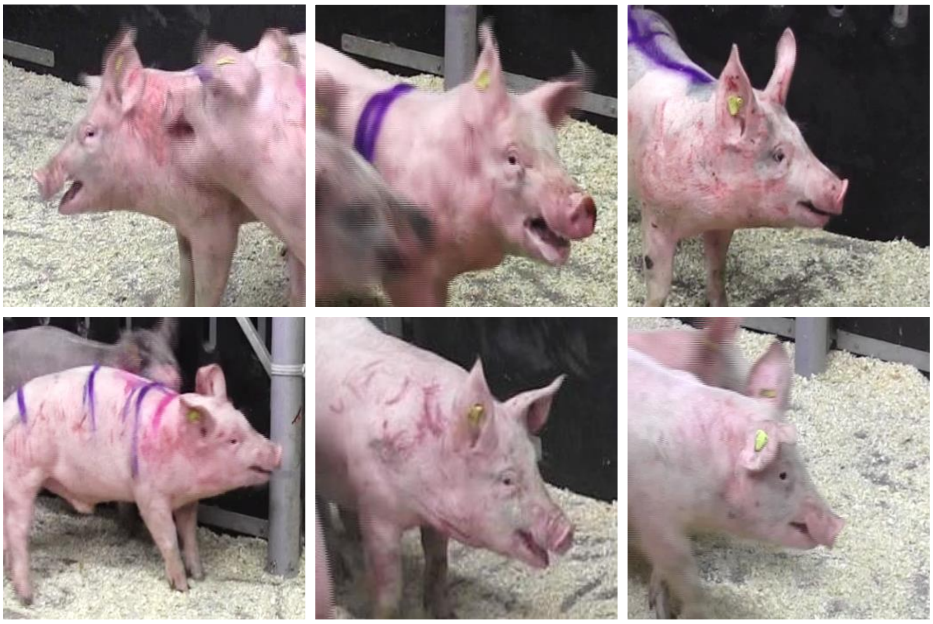

Supplement: Supplementary file 1 — Supplementary file S1, S2 and S3 [file 41598_2018_35905_MOESM1_ESM.pdf]
